# Coronavirus Disease 2019 Vaccine Booster Effects Are Seen in Human Milk Antibody Response

**DOI:** 10.3389/fnut.2022.898849

**Published:** 2022-05-24

**Authors:** Jeffrey M. Bender, Yesun Lee, Wesley A. Cheng, Carolyn J. Marentes Ruiz, Pia S. Pannaraj

**Affiliations:** ^1^Department of Pediatrics, Pediatric Infectious Diseases, Keck School of Medicine, University of Southern California, Los Angeles, CA, United States; ^2^Pediatric Immunization Advancement Laboratory, Children's Hospital Los Angeles, Los Angeles, CA, United States

**Keywords:** breastmilk, breastfeeding, serology, IgA, pregnancy, infant, COVID-19, immunization

## Abstract

Infants remain at high risk for severe coronavirus disease 2019 (COVID-19). Human milk contains high levels of protective SARS CoV-2 specific antibodies post-infection and primary vaccine series, but levels decline over time. We hypothesized that the COVID-19 booster vaccine augment antibody production and the protection afforded to human milk-fed infants. We prospectively enrolled pregnant or lactating mothers planning to receive COVID-19 vaccination. We measured human milk IgG, IgA, and IgM antibodies targeting the SARS CoV-2 receptor binding domain within the spike protein and human milk neutralization activity against SARS CoV-2 in 10 lactating mothers from pre-COVID-19 primary series vaccine to post-booster dose. Human milk SARS CoV-2 specific IgG increased significantly from pre- to post-booster levels (median OD 0.33 vs. 2.02, *P* = 0.002). The IgG levels post-booster were even higher than the peak level after the primary series (2.02 vs. 0.95, *P* = 0.03). The increase in SARS CoV-2 specific IgA levels was not significant (0.10 vs. 0.33, *P* = 0.23). There was a strong correlation between paired maternal blood and milk IgG and IgA levels (IgG rho 0.52, *P* < 0.001, IgA rho 0.31, *P* = 0.05). Post-booster neutralizing activity was elevated compared to pre-booster levels (66% vs. 12% inhibition, *P* = 0.002). COVID-19 vaccine booster elicits SARS CoV-2 specific antibodies in human milk at higher levels compared to the initial primary series. This finding suggests that three doses of COVID-19 mRNA vaccination leads to improved mucosal response in human milk and reinforces current guidance recommending all pregnant or lactating mothers receive full COVID-19 vaccine courses with a booster dose.

## Introduction

As with many other viral illnesses in infants, breastfeeding remains one of the most important ways by which families may protect their newborn children from infection with severe acute respiratory syndrome coronavirus 2 (SARS CoV-2) (1). Epidemiologic surveys initially showed a disproportionate low impact of associated coronavirus disease 2019 (COVID-19) on infants and young children (2–5). The reason for this is likely multifactorial. Maternal transfer of protective SARS CoV-2 specific antibodies is thought to be a major contributor to this natural protection. Numerous studies have demonstrated that SARS CoV-2 infection of mothers during pregnancy leads to transplacental transfer of neutralizing IgG and production of IgA in breastmilk (6–9). Unfortunately, this relative protection is not complete. Infants with COVID-19 have now been shown to be at risk for increased morbidity and mortality and hospitalization rates have increased worldwide (10–14).

Now as the pandemic continues, efforts to vaccinate pregnant and breastfeeding mothers against SARS CoV-2 are critical to further protect them and their infants. As in natural infection, vaccines appear to provide some level of transferred antibodies from mother to infant both through transplacental transport and breastmilk (6, 15–22). Most USA studies thus far have focused on the standard 2 dose series of the messenger ribonucleic acid (mRNA) COVID-19 vaccines (BNT162b2, Pfizer-BioNTech; mRNA-1273, Moderna) or single dose of the adenovirus based COVID-19 vaccine (Ad26.COV2.S, Janssen/Johnson and Johnson). In November 2021, the Centers for Disease Control and Prevention (CDC) recommended that all adults over the age of 18 receive a booster dose of mRNA COVID-19 vaccines 6 months after completing their primary vaccine regimen (1). We hypothesized that this booster COVID-19 vaccine dose would lead to further antibody production and augment the protection afforded to human milk fed infants. Through this brief report, we demonstrate the subsequent maternal antibody response after booster mRNA COVID-19 vaccine dose in breastfeeding mothers.

## Methods

### Study Design/Participants

We prospectively consented and enrolled pregnant or lactating mothers who planned to receive COVID-19 vaccination. Demographic data including pre-existing conditions and prior infection with SARS CoV-2 was obtained at time of enrollment. Participants with known prior infection with SARS CoV-2 were excluded and confirmed in participating subjects by evaluating pre-vaccine blood and breastmilk for antibody response. This study was reviewed and approved by the institutional review board at Children's Hospital Los Angeles.

### Sample Collection

We collected blood and human milk samples at the following time points: pre-vaccination; 1-, 3-, 6-, and 9-months post-initial vaccine dose, and 1-month post-booster vaccine dose. The pre-booster sample collection was defined as the last timepoint prior to the booster vaccination at 6 or 9 months. Individuals received booster doses following emergency use authorization; this occurred between 6 and 9 months for the individuals in our study. Human milk was collected at each time point until the participant stopped lactating. Post booster milk samples were collected between 26-38 days after booster dose. Human milk samples were self-collected at home just prior to each visit in sterile containers or collection bags using electrical or manual pumps. Milk samples were then stored at−80 degrees Celsius (°C) until antibody testing was performed. Blood samples (3-4mL) were drawn during each visit in red top tubes. Blood samples were transported to the laboratory within 2 h of collection where serum was extracted from coagulated blood *via* centrifugation and stored overnight at−20°C for next day serology testing.

### SARS CoV-2 Specific Serology Testing in Human Milk

Measurement of human milk IgG, IgA, and IgM antibodies targeting the SARS CoV-2 receptor binding domain (RBD) within the spike protein was performed using a modified enzyme-linked immunosorbent assay (ELISA) technique (6, 23). In brief, to remove cells and fat, thawed human milk samples were centrifuged at 1,000 G for 10 min twice. The separated supernatant was then diluted to 1:10 and placed in high binding 96-well plates that were previously coated with recombinant SARS CoV-2 RBD protein. These plates were then incubated for 2 h at room temperature. Plates were then washed with PBS-1% Tween20 (PBS-T). Next, diluted (1:3000) secondary enzyme labeled antibodies for IgG, IgA, and IgM (Rockland) were added and incubated for 1 h. We then added 100 uL of O-phenylenediamine dihydrochloric marker substrate (Sigma-Aldrich) to each well and incubated for 20 min prior to quenching with 50 uL of 3 molar hydrochloric acid. Optical density values were measured at 490 nm (OD_490_). We established positive cutoff values based on the mean plus three standard deviations of 20 archived negative control human milk samples collected before 2020 (IgG 0.20, IgA 0.21, and IgM 0.14). The assays were performed in duplicate.

### SARS CoV-2 Specific Serology Testing in Blood

Serum IgG and IgA antibodies targeting the SARS CoV-2 RBD were also measured using a previously described technique (23). All samples were analyzed on the same plate for each isotype assay. We further tested the level of IgG against SARS CoV-2 nucleocapsid protein (GenScript) in pre- and post-booster blood samples of each participant to determine if any SARS CoV-2 infection occurred in the subjects before or during the study.

### Neutralizing Antibody

We measured human milk neutralization activity against SARS CoV-2 using a surrogate virus neutralization assay (sVNT, GenScript). This assay has been previously shown to correlate with the SARS CoV-2 90% plaque reduction neutralization test titer assay (24). We modified this assay as described previously to work with human milk (6). In brief, human milk was mixed with an equal volume of horseradish peroxidase conjugated to recombinant SARS CoV-2 RBD protein and incubated at 37°C for 30 min. Next, 100 uL of each mixture was then added to microtiter plate wells coated with angiotensin-converting enzyme-2 and incubated at 37°C for 15 min. We then added 100 uL of indicator solution (3,3′,5,5′-tetramethylbenzidine) to each well. The plates were then incubated in the dark at 22–25°C for 15 min. Lastly, we added 50 uL of the stop solution and immediately measured the light absorbance at 450 nm. A simple percent inhibition was calculated using negative control values. Using the mean percent inhibition plus three standard deviations of 20 archived negative control human milk samples collected before 2020, we established a cutoff value for neutralization at ≥25% inhibition. The assay was performed in duplicate.

### Data Analysis

Statistical analysis was performed using R Studio v4.0.3 (R Studio). Standard descriptive statistics of median, range, and percent positive based on established cutoffs for each assay at all time points was calculated. Non-parametric variables were then analyzed using Wilcoxon matched-pairs signed-rank tests. Spearman correlation coefficient was used to calculate correlations between serum and human milk values. All tests were designed to be 2-tailed with *P* < 0.05 considered significant.

## Results

### Participants

We identified 10 lactating mothers who we followed and obtained blood and human milk samples pre-vaccine all the way through third booster dose of the COVID vaccine between December 2020 and January 2022 (Table 1). The average age of participating mothers was 35.1 years (range 30.9-42.8). There were few comorbid conditions identified in this cohort with the most common being allergies and obesity. Three were pregnant at time of enrollment and initial vaccination and thus do not have pre-vaccine milk samples. The average gestational age at time of delivery was 38 weeks. Seven of the 10 infants born were female. All mothers reported exclusive breastfeeding at enrollment with the age appropriate introduction of solids as the infants developed. Nine mothers received a full three dose vaccine series with BNT162b2 (Pfizer-BioNTech). One mother was given a single dose Ad26.COV2.S (Janssen/Johnson and Johnson) boosted with a single dose of mRNA-1273 (Moderna). The Ad26.COV2.S individual was included in the primary analysis of pre- vs. post-booster comparisons; she was excluded from the post-primary series analysis to minimize heterogeneity.

**Table 1:** Participant characteristics of 10 lactating mothers receiving the primary series and booster vaccination against COVID-19.

**Characteristics (*n* = 10)**	***N* (%)**
Age, years [mean(range)]	35.1 (30.9–42.8)
**Race**
Asian	2 (20)
White	8 (80)
**Ethnicity**
Non-hispanic	10 (100)
**Highest level of education**
College, Bachelor's degree	4 (40)
Post-graduate degree	6 (60)
**Co-morbid condition**
Allergies	4 (40)
Cancer (past)	1 (10)
Gestational diabetes with last pregnancy, resolved	1 (10)
Other endocrine	1 (10)
Obese (BMI > 30)	3 (30)
Pregnant at enrollment	3 (30)
Gestational age at delivery, weeks [mean, (range)]	38.6 (37.0–39.9)
Infant gender, female	7 (70)
Exclusive breastfeeding at enrollment	10 (100)

### SARS CoV-2 Specific Antibodies Increased After Booster Dose in Human Milk

**Figure 1:**
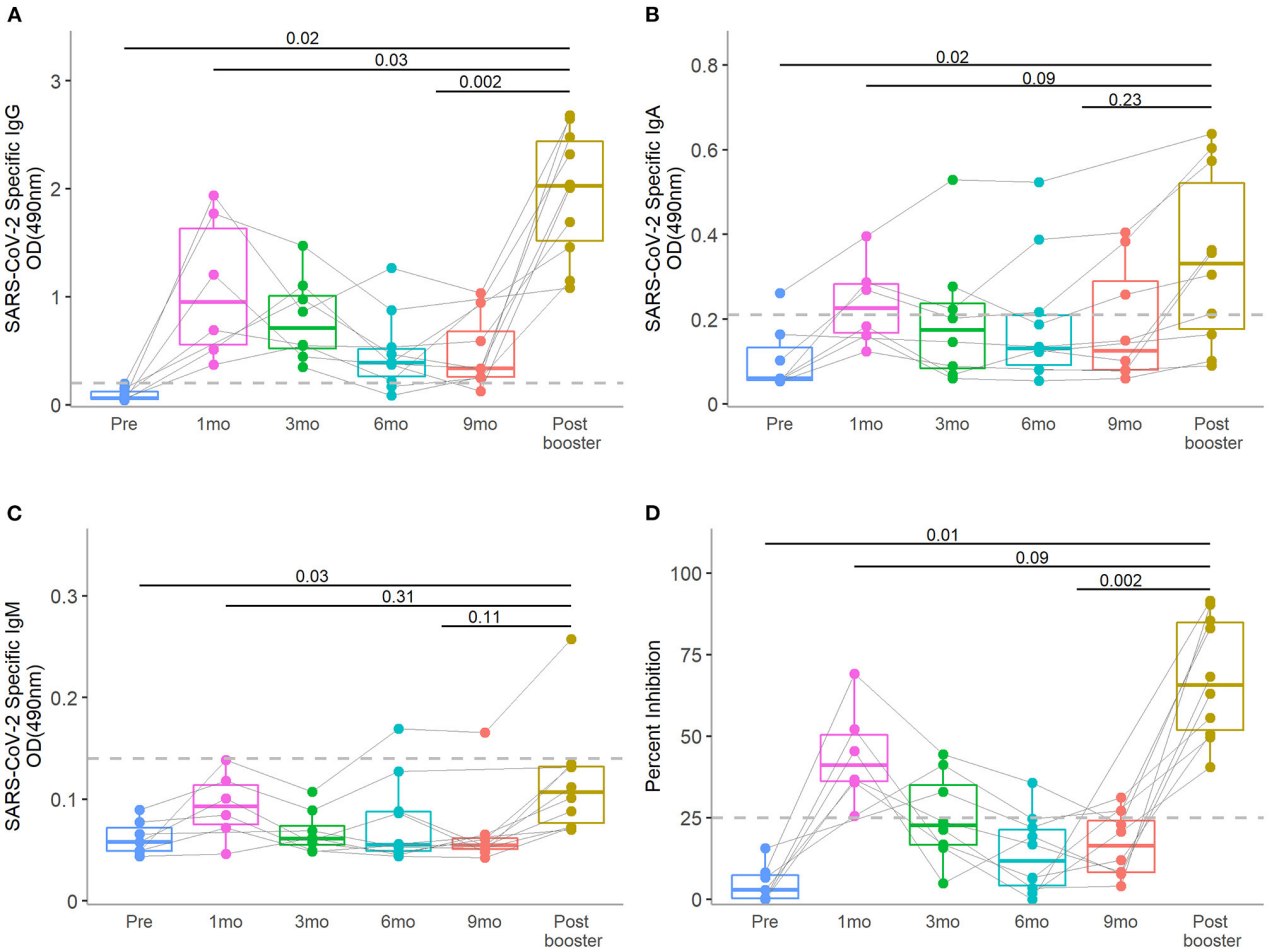
Human milk SARS-CoV-2-specific IgG, IgA, and IgM of antibody levels and neutralizing activity at pre-vaccination: 1-, 3-, 6-, and 9-months post-primary initial vaccine and 1-month post-booster vaccine dose. The median level of SARS-CoV-2-specific IgG, IgA, and IgM and neutralization activity at the 1-month post-booster time point was compared to the peak post-primary vaccination and pre-booster time points in human milk **(A–D)**, respectively]. Dotted lines in y-axis indicate the positive cut-off OD_490_ values of 0.20, 0.21, and 0.13 for IgG, IgA, IgM, respectively. Dotted line in y-axis of **(D)** indicate the positive cut-off of 25% neutralizing activity. Wilcoxon matched pairs signed rank tests were used for statistical analysis. Error bars indicate 95% confidence intervals.

We evaluated the SARS CoV-2 RBD specific antibodies in a total of 48 human milk samples from the 10 lactating mothers (Figures 1A–C, Supplementary Table 1). Pre-COVID vaccine SARS CoV-2 specific IgG, IgA, and IgM levels were all low, suggesting that all participants were immunologically naïve to SARS-CoV-2. Two mothers enrolled at time of first vaccine and one was vaccinated during pregnancy; thus, these three subjects did not provide pre-COVID vaccine milk samples. After the primary vaccine series, SARS CoV-2 specific antibodies increased, peaked at 1 month, and then waned over time. After the booster, human milk SARS CoV-2 specific IgG levels were shown to increase from pre-booster levels (median OD_490_: pre-booster 0.33, post-booster 2.02, *P* = 0.002). The post-booster IgG levels were higher than the initial post primary vaccine series peak (post-primary 0.95, post-booster 2.02, *P* = 0.03). SARS-CoV-2 specific IgA levels showed non-significant increases post-booster compared to pre-booster (pre-booster 0.10, post-booster 0.33, *P* =0.23) and post-primary vaccine series (post-primary 0.23, post- booster 0.33, *P* = 0.09). IgM levels demonstrated little change over the study period.

### COVID Vaccine Booster Led to an Increase in Neutralizing Activity in Human Milk

As seen with SARS CoV-2 specific antibody levels, the percent inhibition peaked in human milk 1 month after initial COVID vaccine and waned with time approaching the booster vaccine dose (Figure 1D, Supplementary Table 1). Post-booster neutralizing activity increased compared to pre-booster levels (median pre-booster 12%, post-booster 66%, *P* = 0.002). The booster vaccine dose led to a non-significant increased neutralizing effect over the peak 1-month post-primary vaccine peak (post-primary 41%, post- booster 66%, *P* = 0.09). All 10 samples collected post-booster demonstrated neutralization with >25% inhibition.

### SARS CoV-2 Specific Antibodies in Human Milk Correlated With Paired Blood Samples

**Figure 2:**
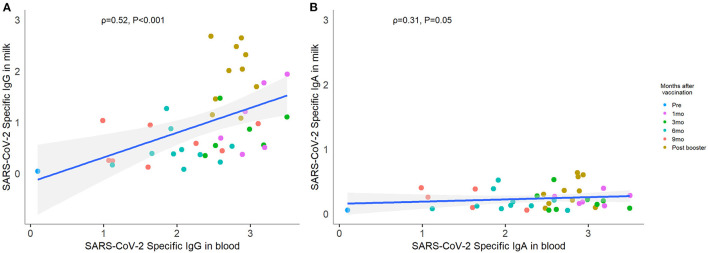
Correlation between paired human milk and blood SARS-CoV-2-specific antibody. Forty-one paired human milk and blood samples collected at the same time point were included in the correlation analysis. Each color point represents each visit. The level of SARS-CoV-2-specific IgG **(A)** and IgA **(B)** in breastmilk showed positive correlations with the same isotypes in blood. Correlations were computed using Spearman correlation coefficient labeled ρ.

In comparing blood SARS CoV-2 RBD specific antibodies to those found in human milk collected at the same time point, we found a moderate correlation (IgG correlation coefficient ρ 0.52, *P* < 0.001; IgA ρ 0.31, *P* = 0.05; Figure 2). As blood levels rose with initial vaccine, both IgG and IgA were similarly elevated. That pattern persisted during the subsequent waning of antibody and post-booster spike. No participants tested positive for COVID-19 during the study period. Furthermore, we confirmed that all pre- and post-booster blood samples were IgG negative against SARS CoV-2 nucleocapsid protein. Therefore, we ensured that all responses observed were due to vaccination.

## Discussion

Through this study, we described that COVID-19 vaccine booster elicits human milk antibody response. SARS CoV-2 specific antibodies (IgG and IgA) increase in human milk after booster dose of mRNA COVID-19 vaccine to levels even higher than the peak after the initial vaccine series. Human milk antibodies boosted with COVID-19 vaccines were further found to have increased neutralizing activity compared to the waning pre-booster activity. The responses correlated with the paired serological blood samples from the lactating mothers. All these findings reinforce current guidance recommending all pregnant or lactating mothers receive full COVID-19 vaccine courses with a booster dose.

Most respiratory infections disproportionately affect infants with a developing immune system (25). Although initial data suggested that infants and children were less likely to acquire SARS-CoV-2 (3–5), increasing rates of pediatric SARS-CoV-2 infections and hospitalizations have been observed worldwide (12–14). Maternal vaccination during pregnancy leads to transplacental antibody transfer (6–9). Breastfeeding may be another important strategy to protect infants. We and others have demonstrated that SARS-CoV-2 specific antibody in human milk following maternal vaccination with the primary series was followed by a slow wane in antibody levels as is seen in natural infection (6, 15–22). Through this study, we observe a clear increase in human milk IgG, IgA, and neutralizing antibody following COVID-19 booster vaccination. To our surprise, the booster dose induced antibody levels even greater than levels generated by the initial vaccination series. This suggests that three doses of mRNA vaccination may provide the optimal mucosal response. Larger studies on how antibody production in lactating mothers may be augmented with multiple doses of COVID-19 vaccine are needed moving forward.

The ability of human milk antibodies to neutralize potential pathogens provides a barrier of protection for infants as they develop. Secretory IgA and IgG can neutralize viruses at the mucosal surface before infection of epithelial cells occurs (26, 27). Previous studies looking at antenatal influenza vaccine in pregnant mothers have demonstrated subsequent production of influenza specific neutralizing antibodies in human milk (28). Neutralizing antibodies induced by COVID-19 vaccines appear to be the key correlate of protection from COVID-19 in animal and human studies (29, 30). Previous studies have demonstrated this neutralization property of human milk SARS-CoV-2 specific antibodies post-primary vaccine series (6, 15, 18, 21). Through this study, we further observe an increase in the ability of these human milk antibodies to neutralize SARS CoV-2 after the COVID vaccine booster dose. Neutralization appeared to be stronger than the peak seen after initial vaccine series, but the difference was not statistically significant likely due to our small numbers. This emphasizes the importance of not only vaccinating lactating mothers to protect their infants but making sure they receive their booster dose as well.

This study has some limitations. The most important limitation is that this is a real-world observational cohort with a small sample size. Though relatively small, this was a unique opportunity to examine 10 COVID naïve mothers who continued to breastfeed through initial and booster doses of vaccine. Despite the small size, we were able to provide statistically significant evidence of antibody response post-COVID-19 booster vaccine. We observed a higher SARS-CoV-2 specific IgG level post-booster compared with the peak post-primary series level. A larger sample size is needed to determine if differences in IgA and neutralizing activity would reach significance. Second, we acknowledge heterogeneity in the study participants. Some received the primary series while pregnant while others were post-partum. The study primarily included mothers vaccinated using the BNT162b2, Pfizer-BioNTech mRNA COVID vaccine. One mother received the Ad26.COV2.S, Janssen/Johnson and Johnson vaccine boosted by a dose of the mRNA-1273, Moderna vaccine. As more vaccine platforms become available, variability around human milk antibody production and protection will need to be evaluated. Furthermore, changing SARS CoV-2 variants and variant-specific boosters may require re-evaluation. Third, despite showing the neutralizing effect of the stimulated antibody after COVID booster vaccine dose, epidemiological studies will be needed to demonstrate protection in infants. A recent study showed that maternal vaccination with mRNA COVID-19 vaccine during pregnancy is effective against COVID-19 hospitalization among infants <6 months of age (31). How much protection was from transplacental transfer vs. from breastfeeding is not clear.

With the ongoing global pandemic, the CDC has strongly recommended all pregnant and lactating mothers to receive a full course of COVID vaccinations including a booster dose (1). Unfortunately, this remains an at-risk population, and vaccine hesitancy has hampered efforts to immunize and protect these mothers and their infants. Our data provides additional evidence to support maternal COVID-19 booster vaccination, as milk-delivered antibodies could offer breastfed infants additional protection against COVID-19.

## Data Availability Statement

The raw data supporting the conclusions of this article will be made available by the authors, without undue reservation.

## Ethics Statement

The studies involving human participants were reviewed and approved by the Institutional Review Board at Children's Hospital Los Angeles. The patients/participants provided their written informed consent to participate in this study.

## Author Contributions

JB, YL, and PP provided conceptualization and study design. WC and CM recruited and enrolled patients. YL conducted experiments and performed data analysis. JB drafted the initial manuscript draft. JB, YL, and PP critically reviewed and revised the manuscript. All authors approved the final manuscript.

## Funding

This study was partially supported by the National Institute of Allergy and Infectious Diseases through Grant No. U01AI144616-02S1.

## Conflict of Interest

PP has received research funding from AstraZeneca and Pfizer for unrelated studies, consultant fees from Sanofi-Pasteur and Seqirus, and speaker fees from Nestle Nutrition Institute. The remaining authors declare that the research was conducted in the absence of any commercial or financial relationships that could be construed as a potential conflict of interest.

## Publisher's Note

All claims expressed in this article are solely those of the authors and do not necessarily represent those of their affiliated organizations, or those of the publisher, the editors and the reviewers. Any product that may be evaluated in this article, or claim that may be made by its manufacturer, is not guaranteed or endorsed by the publisher.
