# Supplementary material for: Coronavirus Disease 2019 Vaccine Booster Effects Are Seen in Human Milk Antibody Response
Source: Front Nutr. 2022 May 24;9:898849. doi: 10.3389/fnut.2022.898849 (PMC9171392; doi:10.3389/fnut.2022.898849)
Supplement: Supplementary file 1 [file Table_1.docx]

Supplemental Table 1.

| **SARS-CoV-2 specific IgG** |  |  |  |  |  |
| --- | --- | --- | --- | --- | --- |
| visit | count | median | QR_25 | QR_75 | Positive*  [n (%)] |
| Pre-vaccination | 7 | 0.06 | 0.05 | 0.12 | 0 (0) |
| 1mo post-primary initial vaccine | 6 | 0.95 | 0.56 | 1.63 | 6 (100) |
| 3mo post-primary initial vaccine | 8 | 0.71 | 0.52 | 1.01 | 8(100) |
| 6mo post-primary initial vaccine | 10 | 0.39 | 0.26 | 0.52 | 8 (80.0) |
| 9mo post-primary initial vaccine | 7 | 0.33 | 0.25 | 0.46 | 6 (85.7) |
| 1mo post-booster vaccine | 10 | 2.02 | 1.52 | 2.44 | 10(100) |
| **SARS-CoV-2 specific IgA** |  |  |  |  |  |
| Pre-vaccination | 7 | 0.06 | 0.06 | 0.13 | 1(14.3) |
| 1mo post-primary initial vaccine | 6 | 0.23 | 0.17 | 0.28 | 3 (50.0) |
| 3mo post-primary initial vaccine | 8 | 0.17 | 0.08 | 0.24 | 3 (37.5) |
| 6mo post-primary initial vaccine | 10 | 0.13 | 0.09 | 0.21 | 3 (30.0) |
| 9mo post-primary initial vaccine | 7 | 0.10 | 0.08 | 0.20 | 2 (28.6) |
| 1mo post-booster vaccine | 10 | 0.33 | 0.18 | 0.52 | 7 (70.0) |
| **SARS-CoV-2 specific IgM** |  |  |  |  |  |
| Pre-vaccination | 7 | 0.06 | 0.05 | 0.07 | 0 (0) |
| 1mo post-primary initial vaccine | 6 | 0.09 | 0.08 | 0.11 | 0 (0) |
| 3mo post-primary initial vaccine | 8 | 0.06 | 0.06 | 0.07 | 0 (0) |
| 6mo post-primary initial vaccine | 10 | 0.06 | 0.05 | 0.09 | 1 (10) |
| 9mo post-primary initial vaccine | 7 | 0.05 | 0.05 | 0.06 | 1 (14.3) |
| 1mo post-booster vaccine | 10 | 0.11 | 0.08 | 0.13 | 1 (10) |
| **Neutralizing activities** |  |  |  |  |  |
| Pre-vaccination | 7 | 2.90 | 0.20 | 7.45 | 0 (0) |
| 1mo post-primary initial vaccine | 6 | 41.05 | 36.10 | 50.43 | 5 (83.3) |
| 3mo post-primary initial vaccine | 8 | 22.60 | 16.63 | 34.97 | 3 (37.5) |
| 6mo post-primary initial vaccine | 10 | 11.75 | 4.20 | 21.33 | 1 (10) |
| 9mo post-primary initial vaccine | 7 | 12.00 | 8.10 | 24.00 | 1 (14.3) |
| 1mo post-booster vaccine | 10 | 65.65 | 51.93 | 84.83 | 10 (100) |

*The positive cut-off OD_490_ values are 0.20, 0.21, and 0.14 for IgG, IgA, IgM, respectively.  The positive cut-off of the neutralizing activity is at ≥25% inhibition.
